# Ultrasonographic characteristics, genetic features, and maternal and fetal outcomes in fetuses with omphalocele in China: a single tertiary center study

**DOI:** 10.1186/s12884-023-05999-3

**Published:** 2023-09-19

**Authors:** Yanting Que, Meiying Cai, Fang Yang, Qingqiang Ji, Shuqi Zhang, Wenhui Huang, Yashi Gao, Bojing Zhou, Hailong Huang, Hua Cao, Na Lin

**Affiliations:** 1 College of Clinical Medicine for Obstetrics & Gynecology and Pediatrics, Fujian Medical University, Fuzhou, China; 2 Medical Genetic Diagnosis and Therapy Center, College of Clinical Medicine for Obstetrics & Gynecology and Pediatrics, Fujian Key Laboratory for Prenatal Diagnosis and Birth Defects, Fujian Maternity and Child Health Hospital, Fujian Medical University, Fuzhou, China; 3 Department of Gynecology, Fujian Maternity and Child Health Hospital, Fuzhou, China

**Keywords:** Omphalocele, Prenatal diagnosis, Karyotype analysis, Chromosome microarray analysis, Whole exome sequencing, Maternal and fetal outcomes

## Abstract

**Background:**

Patients with omphalocele, a midline abdominal wall defect at the umbilical cord base, have a low survival rate. However, the long-term outcomes of fetuses with prenatally diagnosed omphalocele have scarcely been studied. Therefore, we investigated the ultrasonographic features, genetic characteristics, and maternal and fetal outcomes of fetuses with omphalocele and provided a reference for the perinatal management of such cases.

**Methods:**

A total of 120 pregnant females with fetal omphalocele were diagnosed using prenatal ultrasonography at the Fujian Provincial Maternity and Child Health Hospital from January 2015 to March 2022. Amniotic fluid or cord blood samples were drawn at different gestational weeks for routine karyotype analysis, chromosomal microarray analysis (CMA) detection, and whole exome sequencing (WES). The maternal and fetal outcomes were followed up.

**Results:**

Among the 120 fetuses, 27 were diagnosed with isolated omphalocele and 93 with nonisolated omphalocele using prenatal ultrasonography. Cardiac anomalies were the most observed cause in 17 fetuses. Routine karyotyping and CMA were performed on 35 patients, and chromosomal abnormalities were observed in five patients, trisomy 18 in three, trisomy 13 in one, and chromosome 8–11 translocation in one patient; all were non-isolated omphalocele cases. Six nonisolated cases had normal CMA results and conventional karyotype tests, and further WES examination revealed one pathogenic variant and two suspected pathogenic variants. Of the 120 fetuses, 112 were successfully followed up. Eighty of the 112 patients requested pregnancy termination. Seven of the cases died in utero. A 72% 1-year survival rate was observed from the successful 25 live births.

**Conclusion:**

The prognosis of fetuses with nonisolated omphalocele varies greatly, and individualized analysis should be performed to determine fetal retention carefully. Routine karyotyping with CMA testing should be provided for fetuses with omphalocele. WES is an option if karyotype and CMA tests are normal. If the fetal karyotype is normal and no associated abnormalities are observed, fetuses with omphalocele could have a high survival rate, and most will have a good prognosis.

## Background

Omphalocele is a midline abdominal wall defect at the umbilical cord base characterized by absent skin, fascia, and abdominal muscles. The defect is covered by a three-layer membranous sac comprising amnion, Wharton’s jelly, and peritoneum. Omphalocele is one of the most common congenital abdominal wall malformations and is usually detected during ultrasound scanning in the second trimester. The incidence of omphalocele is approximately 3.38/1000 in fetuses [1, 2] and 1.92/10,000 in neonates [3].

Although omphalocele can occur as isolated anomalies, 35–80% of these defects are associated with other structural abnormalities [4–8]. Associated abnormalities include cardiac defects (e.g., ventricular septal defects, tetralogy of Fallot, or dextrocardia), genitourinary abnormalities (e.g., renal or bladder hypoplasia, multicystic nephropathies, hydronephrosis, or ureteral strictures/duplication/ectopic placement), musculoskeletal disorders, gastrointestinal abnormalities (such as malrotation, intestinal or anal atresia), orofacial clefts, neural tube defects, and diaphragmatic defects [3, 8–10]. Routine fetal echocardiography is recommended in omphalocele cases because of the substantially increased risk of structural cardiac abnormalities [11]. Furthermore, the incidence of omphalocele with chromosomal abnormalities ranges from 10 to 67%, including trisomy 13, 18, and 21, Turner syndrome, and triploidy [12–16]. Omphalocele may also be associated with certain genetic syndromes, such as Beckwith–Wiedemann (BWS), pentalogy of Cantrell, and cloacal hypertrophy [17]. Therefore, cytogenetic analysis should be performed if necessary.

One-year survival rates vary between isolated and non-isolated omphalocele. Patients with isolated omphalocele have a 1-year survival rate of > 90%. Infants with omphalocele and an anomaly are seven times more likely to die in the first year of life and typically have lower survival rates [18], with 75% of deaths generally occurring within the first month. With the development of neonatal surgery, some newborns with omphalocele can obtain good outcomes through timely and effective postnatal intervention. Long-term consequences are critical for parents after diagnosis [19]; hence, questions on long-term morbidity, mortality, and quality of life must be answered with reliable data. However, the literature has limited information on the long-term outcomes of fetuses with prenatally diagnosed omphalocele.

In this study, 120 fetuses with omphalocele diagnosed using prenatal ultrasound were analyzed to investigate the prenatal ultrasound features, genetic etiology, pregnancy outcome, and neonatal prognosis to provide a basis for applying genetic testing in prenatal diagnosis and the clinical management of fetuses with omphalocele.

## Methods

### Study participants

This study enrolled 120 pregnant females who received a prenatal diagnosis of omphalocele via fetal ultrasound at a tertiary care center in the Medical Genetic Diagnosis and Therapy Center of Fujian Maternal and Child Health Hospital between January 2015 and March 2022. The gestational age (GA) range was 11 + 2–37 + 3 weeks, and the maternal age range was 19–45 years. The participants were all Han Chinese. The inclusion criteria were singleton or twin fetuses with omphalocele detected using ultrasonography. This study was approved by the Ethics Committee of Fujian Provincial Maternity and Child Health Care Hospital (approval number 2,014,042). Written informed consent for the clinical study was obtained from all patients and their legal guardian(s).

### Prenatal ultrasonography

GE Voluson E8, Philips iU22, Siemens Acuson Sequoia 512, and S2000 color Doppler ultrasonography (probe frequency 2.0–5.0 MHz) were used to examine pregnant females. The pregnant female was placed in a supine position and, if necessary, in the lateral position. Comprehensive routine ultrasonography of the fetus was first performed, and the sections at different angles of each site were observed from head to foot, including the brain, spine, face, abdomen, limbs, placenta, and amniotic fluid. In patients with suspected omphalocele, horizontal transverse sections of the umbilical port, midsagittal sections, and oblique sections of the umbilical artery of the port in the middle abdomen of the fetus were obtained. The size and shape of the mass, the defect occurrence site, the relationship between the mass and the umbilical cord abdominal wall inlet, and internal echoes were observed, and the ultrasound imaging results were recorded.

### Genetic testing

#### Sample collection

Depending on the GA, amniotic fluid or cord blood samples were collected. Approximately 20–35 mL of amniotic fluid was collected via amniocentesis at a GA of 18–24 weeks, or 1.0–2.5 mL of umbilical cord blood was collected via cordocentesis at a GA of > 24 weeks under ultrasound guidance. Simultaneously, 2 mL of peripheral blood was collected from the pregnant females and their spouses and placed in EDTA anticoagulant tubes for future use. The obtained samples were placed in a refrigerator at 4 °C for temporary storage.

#### Routine karyotyping

Fetal amniotic fluid and umbilical cord blood samples were cultured, colchicine treated, collected, and stained according to the laboratory standard operating procedures of G-banded karyotype analysis technical specifications. Karyotypes were analyzed using G-banding. At least 40 metaphases were counted in each case, and five karyotypes were randomly selected for analysis. Chromosomes were named according to the International System of Nomenclature Criteria for Human Cytogenetics (ISCN2015).

#### Chromosome microarray analysis technology

Genomic DNA was extracted from fetal samples, and the genomic DNA concentration and purity were measured using a micro-UV spectrophotometer. The experimental operation followed the standard operating procedures provided by Affymetrix, including DNA extraction and preparation, digestion, ligation, amplification, purification, fragmentation, labeling signal, hybridization, slide staining, and scanning. The raw data were analyzed using the Chromosome Analysis Suite software matched with the kit. Next, the copy number variation (CNV) results were evaluated using public databases, including DGV (http://dgv.tcag.ca/dgv/app/home), DECIPHER (https://decipher.Sanger.Ac.Uk/), Online Mendelian Inheritance in Man (http://www.omim.org), ISCA (https://www.iscaconsortium.org/), and CAGdb (http://www.cagdb.org/). All detected CNVs were classified as pathogenic, likely pathogenic, variants of uncertain clinical significance (VUS), likely benign CNVs, or benign CNVs according to the American College of Medical Genetics standards and guidelines [20]. For VUS results, parents of fetuses are recommended to draw peripheral blood samples for chromosomal microarray analysis (CMA) detection, combined with pedigree analysis, to elucidate the nature of CNV.

#### Whole exome sequencing

Genomic DNA was extracted from fetal samples, genomic libraries were constructed by cutting millions of small DNA fragments, and exotic sequences were obtained using targeted hybridization probes to sequence the DNA. Potential pathogenic homozygous and compound heterozygous variant proteins were selected through bioinformatics analysis of whole exome sequencing (WES) assay data as follows:

(1) Filtering and screening against the human gene mutation database (http://www.hgmd.cf.ac.uk/ac/index.php), National Center for Biotechnology Information (NCBI) single nucleotide polymorphism array (http://www.ncbi.nlm.nih.gov/snp), and analytical procedures that automatically ignore small allele frequencies > 0.01 (including variants in the exome variant server); (2) excluding non-coding variants predicted by the Berkeley Drosophila Genome Project that do not alter splice sites (i.e. http://www.fruitfly.org/seq-tools/splice.html); (3) excluding synonymous variants that do not alter amino acids; (4) excluding variants whose corresponding phenotypes are inconsistent with the genetic characteristics and clinical phenotypes of the proband. Variants detected using whole-exon technology were classified as pathogenic mutations, likely pathogenic mutations, mutations of unknown significance, likely benign mutations, and benign mutations according to the guidelines of the American College of Medical Genetics and Genomics [21].

#### Pregnancy outcome evaluation and follow-up

All pregnant females were followed up via telephone for pregnancy outcomes and fetal postnatal conditions. Follow-up included examination of postnatal physical growth and neurobehavioral development. All adverse pregnancy outcomes, such as stillbirth, dysplasia, and neonatal and infantile deaths, were also followed up. The follow-up was performed until February 10, 2023, with a maximum continuation of 6 years and 7 months.

## Results

### Prenatal ultrasonography

**Fig. 1:**
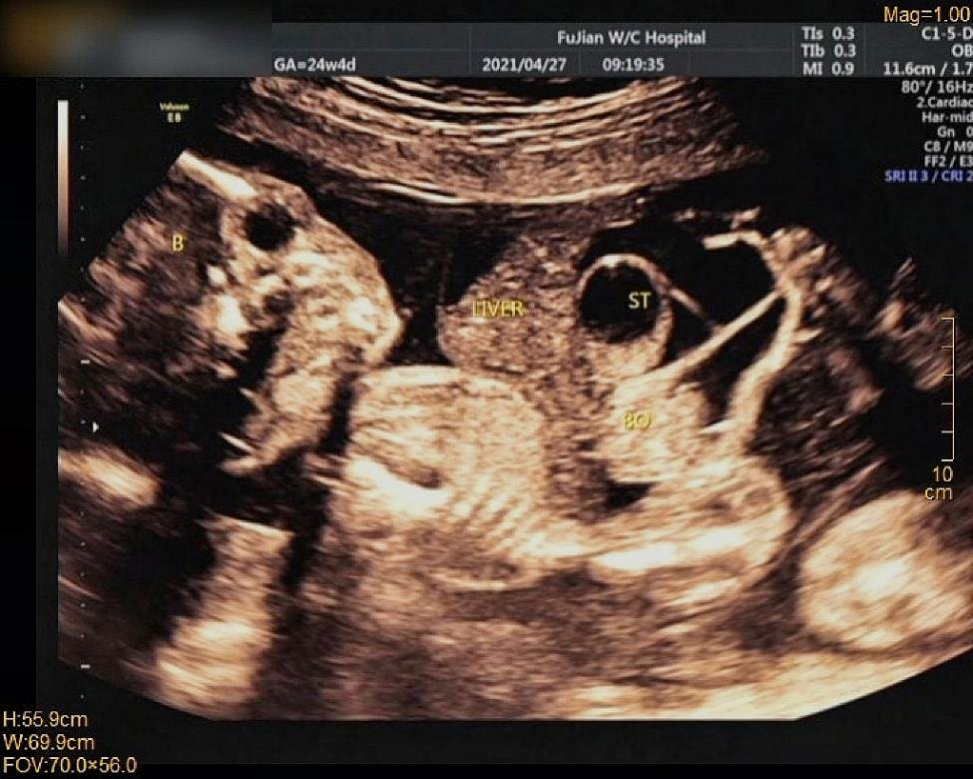
UItrasound image of a fetus at 24 + 4 weeks gestation. Omphalocele containing the liver and bowels. The stomach is intra-abdominal

Among 120 fetuses, 27 cases (22.5%, 27/120) were diagnosed as isolated omphalocele, and 93 (77.5%, 91/120) as nonisolated omphalocele using prenatal ultrasonography. Figure 1 shows an ultrasound image of fetal omphalocele. The most common associated anomaly was a cardiac anomaly combined with structural abnormalities, which was observed in 17 fetuses (54.2%), followed by skeletal (31.2%, 38/120) and central nervous system malformations (22.5%, 27/120). Among omphalocele combined with abnormal soft ultrasound parameters, nuchal translucency thickening (35.9%, 42/120) and reverse a-wave of ductus venosus (29.2%, 35/120) were the most common (Table 1).

**Table 1:** Ninety-three cases of omphalocele with other ultrasound abnormalities

Associated structural anomalies	Number of cases	Percent (%)
Cardiovascular system	65	54. 2
Skeletal system	38	31. 2
Central nervous system	27	22. 5
Facial	23	19. 2
Digestive system	7	5. 8
Urinary system	6	5. 0
Two or more deformities	56	46. 7
Associated ultrasound soft index abnormal	Number of cases	Percent (%)
NT thickening	42	35.9
Venous catheter a-wave reverse	35	29.2

Among the 120 cases, six fetuses with normal CMA and conventional karyotype test results received WES (Fig. 1). The WES results showed three fetuses with normal results and three with abnormal results (Table 2)

### Genetic testing analysis

**Fig. 2:**
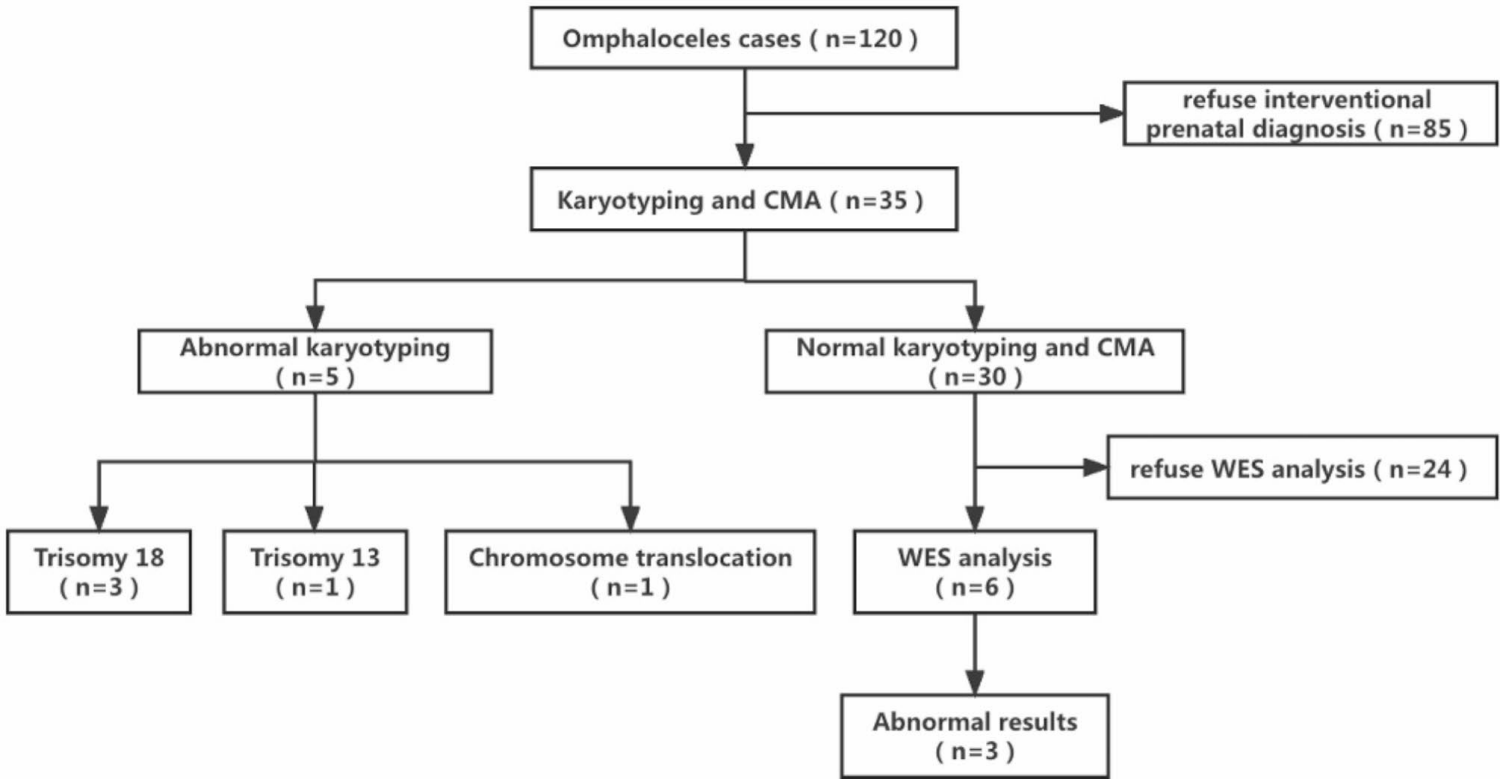
Prenatal diagnosis for fetuses with omphalocele according to genetic analysis type

Among the 120 fetuses, 35 cases (29.2%, 35/120) underwent genetic testing, of which eight (8/33, 24.2%) with isolated omphalocele had normal CMA and conventional karyotype test results. Among the 27 cases (77.1%, 27/35) with nonisolated omphalocele, three fetuses were diagnosed with trisomy 18, one with trisomy 13, and one with chromosome 8–11 translocation. In addition, six fetuses with normal CMA and conventional karyotype test results underwent WES (Fig. 2). The WES results showed three fetuses had normal results and three had abnormal results (Table 2). WES detected the *COL2A1* gene in proband 2759 C > A (p. Pro920His), a heterozygous, likely pathogenic variant. WES also revealed the *SCP2* gene in proband c.674 + 1G > C, a heterozygous, likely pathogenic variant. One case of prenatal ultrasound revealed omphalocele, an absence of the left limb, and WES revealed the *SDHB* gene in proband c. 725G > A (p.R242H), a heterozygous, pathogenic variant.

**Table 2:** Whole exome sequencing (WES) results and pregnancy outcomes of three cases with abnormal results

No.	Clinical information	Gene	Variation	Mode of inheritance	Origin	Variation Rating	Pregnancy Outcome	Postnatal follow-up
1	Prenatal ultrasound revealed omphalocele; fetal anasarca; cardiac dysplasia; absence of main pulmonary artery	COL2A1	Chr12:48372516 NM001844.4 c. 2759 C > A (p. Pro 920 H is) Het	AD	Parent Source	LP	TOP	-
2	Prenatal ultrasound revealed omphalocele and NT thickening.	GLDC	Chr9:6565391 NM000170.2 c. 1889 G > A (p. Arg 640G ln) Het	AR	Maternal origin	VUS	Term delivery	Postnatal neonatal pneumonia, hyperlactatemia, congenital crooked mouth crying syndrome, patent ductus arteriosus. Height and weight loss, mental retardation, rehabilitation
			Chr9:6587220 NM000170.2 c. 1771T > C (p. T yr 591 H is) Het		Parent Source	VUS		
		CTCF	Chr16:67650729 NM006565. 3 c. 1034 A > G (p .His 345 A rg) Het	AD	Newborn	VUS		
		SPTAN1	Chr9:131356645 NM001130438. 2 c. 3407T > A (p. Phe 1136T yr) Het	AD	Parent Source	VUS		
		SCP2	Chr1:53442442 NM002979. 4 c. 674 + 1G > C Het	AR	Maternal origin	LP		
3	Prenatal ultrasound revealed omphalocele and absence of left limb	SDHB	Chr1:1734914 3 NM003000.3 C.725G > A (p.R242H) Het	AD	Maternal origin	P	TOP	-

P, pathogenic; LP, likely pathogenic; VUS, variants of uncertain significance. Pattern of inheritance: AD (autosomal dominant), AR (autosomal recessive)

### Pregnancy outcomes

All 120 omphalocele cases were followed up to assess the pregnancy outcome and postnatal development. Only 112 cases were successfully followed up, with a follow-up rate of 93.3% (112/120). Among the 112 successfully followed cases, 80 (71.4%, 80/112) terminated the pregnancy (6.3%, 7/112), and seven intrauterine fetal deaths were observed, leaving 25 (22.3%, 25/112) live-born infants (Table 3).

**Table 3:** Outcomes of 112 fetuses with omphalocele

	Induction of labor (n/%)	Intrauterine fetal death (n/%)	Live birth (n/%)	Total
Isolated omphalocele	10 (43. 4)	1 (4. 3)	12 (52.1)	23
Non isolated omphalocele	70 (78. 7)	6 (6. 7)	13 (14. 6)	89
Total	80 (71. 4)	7 (6. 3)	25 (22. 3)	112

### Postnatal conditions

Among the 25 live births, 10 neonates (40%, 10/25) underwent conservative treatment, nine (36%, 9/25) underwent pediatric surgery, and six (24%, 6/25) abandoned treatment or died due to poor prognosis. Seven infants (28%, 7/25) died in the first year. Further details regarding the clinical characteristics of the infants with omphalocele who died postnatally are presented in Table 4. During follow-up to 1 year of age, 18 neonates survived, with a 1-year survival rate of 72% (18/25). The youngest was 1 year, and the oldest was 6 years and 7 months. Of the 18 surviving neonates, 33% had subnormal growth parameters, including six (33.3%, 4/18) with weight below the 10th percentile and four (22.2%, 4/18) with height below the 10th percentile. Gastrointestinal disorders were present in 27.8% of patients, and chronic abdominal pain was present in 11.1%. Two patients (11.1%, 2/18) had language delay, and one (5.6%, 1/20) had mental disabilities; both received rehabilitation (Table 5).

**Table 4:** Clinical characters of the infants with omphalocele who died postnatally

G A at birth (weeks)	Age at death (days)	Characters	Cause of death
35 + 6	2	Giant omphalocele; bladder exstrophy; rectovesical or colovesical fistula; anal atresia (high position); hypospadias; bilateral abdominal cryptorchidism	Abandoned treatment
40 + 1	3	Neonatal trisomy 18; cleft lip and palate; polyarticular contractures; omphalocele; severe neonatal asphyxia	Abandoned treatment
32	3	Small for gestational age; cleft lip and palate; polyarticular spasm; pulmonary dysplasia	Respiratory failure
30 + 4	1	Giant omphalocele; small for gestational age; bilateral cryptorchidism; myocardial damage; coagulation abnormalities; heart failure; shock; neonatal pneumonia	Respiratory failure
39	3	Small for gestational age; neonatal pneumonia; anal atresia; absence of left kidney; scoliosis	Abandoned treatment
34	17	Giant omphalocele; lung dysplasia; sepsis	Respiratory failure
37	33	Fetus A normal, Fetus B giant omphalocele	Fetus B performed surgery after birth and presented with jaundice and pulmonary edema 1 month after surgery

**Table 5:** Clinical features and outcomes of the 18 surviving fetuses with omphalocele

		Found GA (weeks)	Prenatal Ultrasound Results	Surgical/Conservative	Age	Follow-up Results
Isolated omphalocele	1	12 + 1	Omphalocele	-	-	Healthy
	2	12 + 5	Omphalocele	-	-	Healthy
	3	12 + 6	Omphalocele	-	-	Healthy
	4	23 + 3	Omphalocele	Excision of umbilical fistula + omphaloplasty	2 Years	Low body weight; Gastrointestinal disorder
	5	21 + 6	Omphalocele	Omphalocele repair	1 year 1 month	Healthy
	6	11 + 2	Omphalocele	Conservative	2 years 2 months	Healthy
	7	12 + 4	Omphalocele	Conservative	4 Years	Language delay, rehabilitation
	8	13 + 1	Omphalocele	Conservative	4 years 11 months	Healthy, postnatal patent foramen ovale
	9	37 + 3	Omphalocele	Conservative	5 years 11 months	Healthy
	10	13	Omphalocele	Conservative	6 Years	Healthy, slightly bulging umbilicus
	11	12 + 6	Omphalocele	Conservative	6 years 7 months	Healthy
Nonisolated omphalocele	12	12 + 5	Omphalocele; NT fetal thickening	Conservative	3 years 1 month	Postnatal neonatal pneumonia, hyperlactatemia, congenital crooked mouth crying syndrome, patent ductus arteriosus. Mental retardation, growth retardation, rehabilitation
	13	12 + 4	A fetus was normal; B fetus had omphalocele, mild dilatation of the bowel, and mild dilatation of the right renal pelvis	Congenital omphalocele repair + omphaloplasty + omphaloenteric fistula resection + intestinal anastomosis + intestinal adhesiolysis + right lower quadrant drainage	4 years 2 months	Low height and weight; gastrointestinal disorders; language delay, rehabilitation
	14	14 + 2	Omphalocele; fetal measurements less than weeks of menopause; accessory renal artery of left kidney	Omphalocele repair	1 year	Healthy
	15	23 + 6	Omphalocele, mild separation of fetal left renal pelvis	Repair of congenital omphalocele + omphaloplasty	1 year 1 month	Healthy
	17	12 + 3	Omphalocele, venous catheter a-wave reversal	Repair of omphalocele + intestinal adhesiolysis + appendectomy	3 years 4 months	Low height and weight, normal mental capacity; gastrointestinal dysfunction; intestinal obstruction surgery at the age of 2
	17	10 + 6	Omphalocele, patent heart and	Omphalocele reposition, repair + umbilical plasty	4 years 10 months	Low height and weight; Gastrointestinal disorders; chronic abdominal pain
	18	24 + 5	Omphalocele; mild separation of bilateral renal collecting systems; mild separation of fetal bilateral renal collecting systems; abnormal blood flow spectrum of fetal middle cerebral artery and umbilical artery	Omphalocele repair	3 years 2 months	Ectrodactyly; underweight; gastrointestinal disorders; chronic abdominal pain

## Discussion

In this study, 120 fetuses with ultrasound-confirmed omphalocele were analyzed to investigate the prenatal ultrasonographic features, genetic etiology, and maternal and fetal outcomes of fetuses with omphalocele and provide a reference for perinatal management of these cases. Most omphalocele cases were combined with other abnormalities detected using ultrasound. The most common abnormality was cardiovascular system malformations, consistent with a multicenter study of omphalocele cases from 2005 to 2013 [22]. In contrast, a multicenter database of omphalocele cases from 1997 to 2012 reported a 35% incidence of associated congenital anomalies [5]. The differences may be related to different instruments used in the study group, operator skill level, and screening mechanisms at different periods. The risk of chromosomal abnormalities in fetuses with omphalocele varies according to maternal age, GA at diagnosis, malformations, and omphalocele contents. In this study, the karyotype abnormality detection rate in 35 fetuses with omphalocele was 14.3%, consistent with the results of other studies [12–16].

Omphalocele suggests an increased risk of aneuploidy [23]. A first-trimester study analyzed clinical data from 98 children with omphalocele, and 53.8% of fetuses had chromosomal abnormalities. The most common aneuploidy was trisomy 18, and 78.9% of the children had other systemic malformations [24]. This finding suggests that chromosomal abnormalities considerably increase when omphalocele is associated with other systemic malformations. This study detected three cases of trisomy 18, one of trisomy 13, and one of chromosome 8–11 translocation in fetuses with nonisolated omphalocele. Therefore, when a prenatal ultrasound suggests omphalocele, systematic structural screening of the fetus should also be performed to determine whether it is associated with other structural abnormalities, along with genetic testing to identify chromosomal abnormalities.

CMA technology enables scanning at the genome-wide level and can detect unbalanced rearrangements, such as minor deletions and duplications in the chromosome [25]. The additional detection rate of CMA for fetal structural abnormalities suggested through ultrasound can reach 6.0–8.0% [25]. However, owing to the small number of omphalocele cases included in the prenatal study, the exact utility of CMA remains unclear. CMA was performed in 81 children with omphalocele, and one pathogenic CNV was detected, with a 1.2% detection rate [26]. In our study, CNV was not found, possibly due to the limitation of the abnormal ultrasound phenotype and the small sample size. However, the most common genetic cause found was aneuploidy rather than CSV.

When karyotype and CMA results are normal, using WES as the next step may increase the diagnostic yield in fetuses with structural abnormalities by approximately 8–10% [27]. Our study used WES to identify six cases of nonisolated omphalocele. Routine karyotype analysis and CMA detection were normal in three probands, and WES detected three abnormalities. One prenatal ultrasound revealed omphalocele, fetal anasarca, cardiac dysplasia, and absence of the main pulmonary artery. WES detected a heterozygous, possibly pathogenic variant in the proband that was inherited from the father. Heterozygous mutations in the *COL2A1* gene are often associated with a spectrum of dwarfism and skeletal malformations [28]. One prenatal ultrasound revealed omphalocele and NT thickening, whereas WES revealed the *SCP2* gene inherited from the mother and associated with leukodystrophy with dystonia and motor neuropathy [29]. Three VUS were found in the proband that have not been documented and need further investigation. Furthermore, the S*DHB* gene detected through WES is inherited from the mother and is associated with hereditary paraganglioma-pheochromocytoma syndromes (PGL/PCC syndromes) (OMIM 115,310). PGL/PCC syndromes have variable clinical manifestations, with clinical phenotypes including paragangliomas and their complications (such as pulsatile tinnitus, conductive hearing impairment, cranial nerve palsy, hoarseness, and vocal cord paralysis), pheochromocytomas, and multiple organ dysfunction associated with elevated catecholamines (such as palpitations, tachycardia, hypertension, hyperhidrosis, headache, and anxiety) [30–32]. However, the literature did not mention omphalocele, whereas the ultrasound phenotype of the fetus in this study had omphalocele, indicating that a c. 725G > A (p.R242H) variant may be associated with omphalocele development. Therefore, WES can be used as a further test for fetuses with nonisolated omphalocele when karyotype and CMA cannot provide a diagnosis.

Omphalocele may also be associated with certain genetic syndromes [17]. BWS is one of the most common overgrowth syndromes and is clinically characterized by macroglossia, macrobodies, umbilical hernia, abdominal wall defects, omphalocele, abdominal organomegaly, neonatal hypoglycemia, and earlobe line fractures. Abnormal regulation of imprinted gene expression in the chromosome 11p15.5 region can cause the BWS phenotype, and approximately 80% of BWS cases have molecular changes in chromosome 11p15, partially due to a somatic mosaic of molecular changes. Multiple mechanisms can cause BWS through epigenetic or genetic alterations in the chromosome 11p15.5 imprinting domain. Such changes include parent-specific duplications, translocations or inversions, microdeletions, microduplications, altered DNA methylation at imprinting centers 1 or 2, and uniparental disomies, which alter the relative contribution of parental alleles [33–35]. BWS can be diagnosed via molecular testing, and methylation-sensitive multiplex ligation probe analysis is the most reliable clinical test for most epigenetic and genetic BWS causes [36]. BWS occurs in 2–25% of fetuses diagnosed with omphalocele [37, 38]. However, one BWS case (0.8%, 1/120) alone was detected in our study; the potential reason for this difference is that fetuses with omphalocele typically do not undergo prenatal care for BWS at our institution. Another possible reason is that some BWS features develop in the third trimester or postpartum period, and most parents choose to terminate the pregnancy without further genetic testing after multiple malformations. Therefore, when fetal prenatal ultrasound suggests omphalocele, the karyotype, CMA, and WES analyses cannot confirm the diagnosis. Hence, BWS-related detection methods should be further performed in clinical practice.

Among the cases successfully followed in this study, the live birth rate was 22.3%. The infant mortality rate in our study was 28.0%, and 85.7% occurred in the first days of life. However, an Australian study reported a slightly lower neonatal mortality rate of 15.6% [39], whereas we observed a mortality rate of 24.0%. One-year survival rates vary between isolated and nonisolated omphaloceles, with isolated omphaloceles having a better pregnancy outcome and often having a high survival rate of 90% [3]. Among the cases we followed, one prenatal karyotype showed trisomy 18, and the prenatal diagnostic center recommended inducing labor. The patient and her family chose to continue the pregnancy, delivering a fetus at 40 + 1 weeks with a cleft lip and palate at birth, polyarticular contractures, omphalocele, and severe asphyxia, who died 3 days after birth. The survival rate of 1-year-old infants in our study was 72%, including 91.7% for isolated omphalocele, similar to a US study from 1995 to 2005 [3], which showed that a 22% increase in the one-year survival rate was observed from 2001 to 2005 compared to the five years before. This finding may indicate that newborns with omphalocele receive better prenatal diagnosis or treatment. Recent studies suggest that many termination of pregnancy-eligible couples choose to continue their pregnancies. The focus of antenatal care should be on maternal health, not fetal survival, and follow-up plans should aim at timely diagnosis and effective management of obstetric complications as part of the counseling provided to the couple [40].

Growth is compromised in infants with omphalocele, with 39–65% of children reporting birth weight below the 10th percentile, similar to our results [41, 42]. Gastrointestinal disorders and chronic abdominal pain were observed in patients in this study. Van Eijck et al. [43] reported similar results (27%); however, their older patient group suggested that chronic abdominal pain plays an important role in childhood and early adulthood, requiring further follow-up. In this study, two patients had a delay in speech development, and one had mental disabilities. However, in a Dutch collaborative survey., 77% of children born with omphalocele eventually received post-secondary education, and the proportion is considered similar to the national population [43]. Over 93% of children with a history of omphalocele attended kindergarten at the expected age [8]. These studies showed that despite initial difficulties, most children with omphalocele eventually achieve age-appropriate neurodevelopment. However, the impact of omphalocele on neurological development remains unclear as most studies have selection bias and underlying diseases (genetic or structural abnormalities), patient-specific risks (preterm birth or birth weight), and environmental factors associated with neonatal and perioperative management, and nutritional status [44] may play an important role. Neurological and mental development is a complex multifactorial process, and only severely affected children can benefit from long-term follow-up, requiring data from larger cohorts and longer follow-up periods.

This study had some limitations. First, the sample size was limited, and only 120 fetuses with omphalocele were retrospectively analyzed. Second, no information was provided regarding the omphalocele size or delivery mode. In addition, only some cases were detected via karyotyping, CMA, and WES, and the chromosomal causes of partial omphalocele were not excluded.

In conclusion, the prognosis of fetuses with nonisolated omphalocele varies greatly, and individualized analysis should be performed to determine fetal retention carefully. This study provides more phenotypes associated with omphalocele that can be added to the databases. Routine karyotyping with CMA testing should be offered because of the increased risk of fetal aneuploidy due to prenatal omphalocele, specifically if combined with other ultrasound abnormalities. WES is an option if karyotype and CMA tests are normal. In addition, if the results of conventional karyotype, CMA detection, and WES detection are normal, further molecular biology methods can be performed to rule out disease phenotypes like BWS. If the fetal karyotype is normal and no associated abnormalities are observed, fetuses diagnosed with omphalocele will have a higher survival rate, with the majority having a good prognosis and blind pregnancy termination is not recommended. Other malformations adversely impact perinatal mortality because of the increased risk of intrauterine death and immediate neonatal death in fetuses with omphalocele. In the future, more omphalocele cases should be investigated, and karyotype analysis, CMA, and WES detection should be performed further to improve the perinatal management of fetuses with omphalocele.

## Data Availability

Data and materials are available upon reasonable request. The data and materials supporting this study’s findings are available from the corresponding authors upon reasonable request. The data discussed in this publication have been deposited in NCBI’s Gene Expression Omnibus (Que et al., 2023) and are accessible through GEO Series accession number GSE235430 (https://www.ncbi.nlm.nih.gov/geo/query/acc.cgi?acc=GSE235430).

## List of Abbreviations

AD: Autosomal dominant
AR: Autosomal recessive
BWS: Beckwith–Wiedemann
CMA: Chromosomal microarray analysis
CNV: Copy number variation
GA: Gestational age
LP: Likely pathogenic
NCBI: National Center for Biotechnology Information
PGL/PCC: Paraganglioma-pheochromocytoma syndromes
P: Pathogenic
VUS: Variants of uncertain significance
WES: Whole exome sequencing
